# Serial Systemic Immune-Inflammation Indices (SSIIi) as Prognostic Markers in Persistent Hypoxic Brain Injury Post-cardiac Arrest: A Case Report

**DOI:** 10.7759/cureus.67299

**Published:** 2024-08-20

**Authors:** Tissa Wijeratne, Sheila G Crewther, Chanith Wijeratne, Richard Shao

**Affiliations:** 1 Department of Neurology, Western Health, La Trobe University, St. Albans, AUS; 2 Department of Neurology and Stroke Services, Australian Institute for Musculoskeletal Science, Sunshine Hospital, St. Albans, AUS; 3 Department of Psychology and Public Health, La Trobe University, Bundoora, AUS; 4 Department of Neurology, Monash Medical School, Clayton, AUS; 5 Department of Neurology, Sunshine Hospital, St. Albans, AUS

**Keywords:** serial systemic immune-inflammation index (ssiii), systemic immune-inflammation index (siii), hypoxic-ischemic brain injury, neuroinflammation, hibi

## Abstract

This case report presents a novel exploration of serial systemic immune-inflammation indices (SSIIi) as a potential prognostic biomarker in a critical care setting. The subject of this report is a 31-year-old male who, following a heroin overdose, suffered an asystolic cardiac arrest and subsequently passed away two weeks later in the intensive care unit (ICU). The SSIIi, calculated as platelet count × neutrophil count / lymphocyte count, was monitored throughout his stay. The case demonstrates that SSIIi measurements, particularly within the critical initial 24-72 hours, may provide insight into the patient's immune response dynamics following a severe hypoxic event. Specifically, the data suggest that a persistently elevated SSIIi may be indicative of a maladaptive immune response, characterized by ongoing inflammation, which correlates with a deteriorating clinical trajectory. The rapid escalation and sustained high SSIIi values observed in this patient appear to predict a poor outcome.

This case underscores the importance of SSIIi as a potential tool for clinicians to assess prognosis in ICU patients, particularly in cases of acute brain injury where hypoxia is a central factor and sepsis is not present. The findings open avenues for further research into SSIIi as an objective measure for guiding treatment decisions and improving outcomes in similar critical care scenarios.

## Introduction

This case report discusses a 31-year-old male patient with a complex medical history, including chronic back pain, bipolar affective disorder (BPAD), and anxiety. The patient was found by his partner engaging in heroin use in the early hours of the morning. Although initially responsive and showing signs of life, he was later discovered unresponsive, leading to an emergency response. Despite initial interventions, the patient suffered a cardiac arrest and required advanced life support measures. He was subsequently admitted to the intensive care unit (ICU), where he remained in a minimally conscious state with significant neurological impairment. This report outlines the clinical progression, interventions, and outcomes during his ICU stay, with a focus on the neurological and systemic complications that arose as a result of the incident.

## Case presentation

A 31-year-old male with a history of chronic back pain, bipolar affective disorder (BPAD), and anxiety was observed by his partner snorting heroin around 1:00 am. His partner allowed him to continue sleeping after noting that he was alive and snoring at 8:00 am. However, at 10:38 am, she found him unresponsive and likely unconscious, prompting her to immediately call an ambulance and begin cardiopulmonary resuscitation (CPR). The first ambulance crew arrived at 10:59 am and administered 3 mg of intravenous adrenaline while continuing CPR. A second ambulance crew arrived at 11:00 am, observing fully dilated but reactive pupils. The patient developed ventricular fibrillation and cardiac arrest at 11:19 am, after which he was treated with two shocks at 200 joules and an additional 2 mg of intravenous adrenaline. An intravenous adrenaline infusion was initiated, and the patient was transferred to the nearest tertiary hospital intensive care unit (ICU), where he was supported with assisted ventilation and hemofiltration for the next 14 days.

During his ICU stay, the patient remained minimally conscious, with spontaneous eye-opening and an extensor response to painful stimuli. His optic discs were ophthalmologically or ophthalmoscopically normal on admission and during subsequent neurologist reviews. He had no purposeful eye movements, normal limb reflexes, and a bilateral flexor plantar response, with intact brain stem reflexes. Blood tests showed evidence of ischemic injury to multiple organs as detailed in Table 1.

**Table 1:** Summary of key blood tests INR: international normalized ratio, ALT: alanine aminotransferase, GGT: gamma-glutamyl transferase, CPK: creatine phosphokinase

Test	Result	Normal range
Troponin I ( Beckman)	776 ng/L	<21 ng/L
INR	1.7	<1.3
ALT	100,000 U/L	0-55 U/L
GGT	190 U/L	30-110 U/L
Creatinine	245 µmol/L	60-110 µmol/L
Sodium	148 µmol/L	135-145 µmol/L
Potassium	5.5 µmol/L	3.3-4.9 µmol/L
Serum lactate	14 µmol/L	<2.2 µmol/L
CPK	617 U/L	0-240 U/L
C-reactive protein	1 mg/L	0-10 mg/L

A computed tomography (CT) scan of the brain at 72 hours post-admission and a 3 Tesla magnetic resonance imaging (MRI) scan on day 8 revealed no abnormalities such as vascular insults or white matter intensities. An electroencephalogram (EEG) performed on day 7 also showed no significant epileptiform abnormalities. Due to anuric acute renal failure, likely caused by ischemia-induced acute tubular necrosis, hemofiltration was conducted throughout the ICU stay.

On day 12 post-admission, the ICU team sought an independent neurology review to assess the patient, as they believed the prognosis was poor given the high blood test readings indicative of liver and kidney stress and presumably due to persistent severe hypoxic brain injury. Two neurologists reviewed the imaging and electrophysiological data associated with the case and felt that the evidence of severe hypoxic brain injury was insufficient, to offer diagnostic certainty to support the ICU team's poor prognosis.

A third neurology opinion was sought from (TW) regarding the prognosis, as the patient had previously expressed to his partner and family that he did not wish to prolong his life in the event of severe disability. The third neurologist's clinical assessment aligned with the previous neurologists' findings, but he recommended the withdrawal of ventilation and a shift to palliative care on the basis of the routine blood tests (high levels of creatinine and GGT indicative of liver and kidney damage (Table 1)) and systemic immune-inflammation results and the literature indicating that normal CT scan, MRI, and EEG often are not diagnostic of severe hypoxic-ischemic brain injury (HIBI) and poor prognosis [1-3].

**Figure 1:**
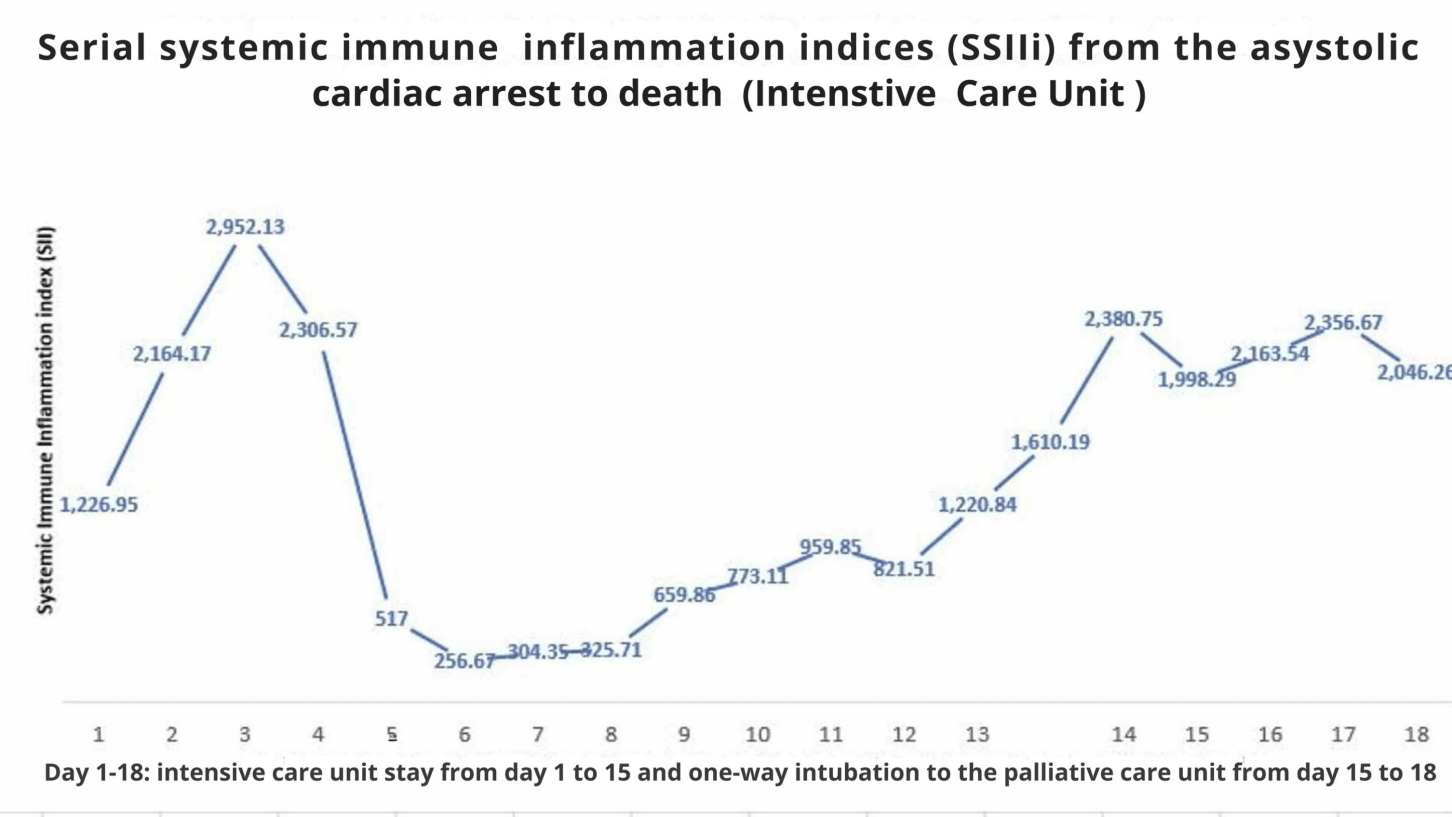
SSIIi from admission to death SSIIi: serial systemic immune-inflammation indices

At this point, the systemic immune-inflammation index (SIIi) for each blood test performed during the hospital stay was manually calculated (Figure 1). The SSIIi was used as an objective prognostic marker of poor outcome in this case, considering that in cancer research, Hu et al. [4] regard an SIIi > 330 as indicative of unlikely recovery. With full agreement among all parties, ICU supportive care and assisted ventilation were withdrawn on day 15, and the patient was transferred to a general medical ward with palliative care support. He passed away peacefully on day 18.

## Discussion

The aim of this case study is to report the use of SSIIi measurements as a potential serum biomarker for poor prognosis in HIBI. As described above, the case involves the prognosis of a 31-year-old male in the ICU who suffered HIBI following a heroin overdose and cardiac arrest with a discussion between intensivists and neurologists arising after two weeks in the ICU regarding the outcome prognosis after HIBI, which was ultimately resolved by serial calculations of SIIi from existing blood tests.

Currently, no widely available objective measure of prognosis exists for patients admitted to the ICU following a community cardiac arrest [4]. Approximately 80% of these patients are comatose upon ICU admission, and around 70% will die due to hypoxic-ischemic brain injury (HIBI) [5-9]. HIBI leads to neuronal death and diffuse brain edema, sharing pathobiology with acute ischemic stroke and COVID-19-related brain disorders. Death in HIBI cases is often due to the withdrawal of life-sustaining treatment after a poor neurological outcome is confirmed, rather than directly from visible cerebral ischemia. Current neurological guidelines for decision-making in the ICU recommend a combination of predictors, including clinical neurological examination, neuroimaging, electrophysiological investigations, and serum biomarkers. However, many of the recommended serum biomarkers are not easily accessible in many countries, including Australia. Alternative blood-based immune predictors are rarely considered, despite their use in other diseases, such as the neutrophil-to-lymphocyte ratio (NLR) in stroke and the systemic immune-inflammation index (SIIi) in various conditions [10].

Thus, our aim was to explore the diagnostic potential of routine blood-based immune cell counts as objective, rapid, and readily available prognostic indicators for use in ICU settings and as objective measures to determine the appropriate time and withdrawal of life-sustaining intervention. Neutrophils are the most abundant white blood cells in peripheral blood and play a critical role in the initial innate immune response to injury (such as acute stroke, hypoxic brain injury, and infections) by releasing inflammatory contents from their granules and attracting other inflammatory cells. Similar immune responses have been observed in the context of COVID-19 and its persistent neurological sequelae. On the other hand, lymphocytes are responsible for the specificity of adaptive immunity, in combination with lymphopenia, neutrophilia, and elevated systemic immune-inflammation index (SII), and appear to correlate with the extent of ischemic injury and the clinical severity of vascular insults.

The serial systemic immune-inflammation index (SSIIi) was initially developed as a measure of chronic inflammation in colon cancer and later explored for its prognostic value in hepatocellular carcinoma (HCC). The SIIi is derived from peripheral blood counts of lymphocytes, neutrophils, and platelets and is calculated as SIIi = platelet count (P) × neutrophil count (N) / lymphocyte count (L) [4]. A single SIIi value over 330 has been significantly associated with larger tumors, early recurrence, and vascular invasion in HCC.

To the best of our knowledge, there are no published reports using SSIIi to predict outcomes in cases of drug overdose-induced cardiac arrest. One report has been found that utilized two SIIi measurements before and 24 hours after thrombolysis in 165 ischemic stroke patients [11]. Wijeratne et al. [12], Wijeratne et al. [13], and Wijeratne et al. [14] described the role of SSIIi in post-COVID-19 neurological syndrome (PCNS) [15], suggesting that further exploration of SSIIi was warranted, as these patterns may offer an objective, systemic biological marker of prognosis across various conditions, including HIBI, stroke, COVID-19-related neurological disorders, vaccination-related adverse effects, and traumatic brain injury.

Conditions such as stroke, hypoxic-ischemic brain injury (HIBI), and COVID-19-related brain disorders are reported to share common elements of pathobiology involving damage-associated molecular patterns (DAMPs) (such as interleukin-1 (IL-1), tumor necrosis factor-alpha (TNF-α), and interleukin-6 (IL-6) when sterile antigens from necrotic neuronal cells are captured by monocytes and dendritic cells). Lymphocytes, responsible for the specificity of adaptive immunity, in combination with lymphopenia, neutrophilia, and elevated systemic immune-inflammation index (SII), appear to correlate with the extent of ischemic injury and the clinical severity of vascular insults.

Currently, the value of serial SII measurements as a tool to estimate benefit for prognosis following stroke has only been investigated by Topcuoglu et al. (2021) [11], who explored the use of single measures of SII before and 24 hours after intravenous tissue plasminogen activator (tPA) treatment to predict functional outcomes and the risk of tPA-related symptomatic intracerebral hemorrhage and found the index to be only moderately sensitive and specific. However, we propose that the true usefulness of SII lies in serial measurements during the acute stage of illness where such measurements can serve as a first-line objective measure of prognosis and, over time, as a consistent indicator of the immunological response to prolonged physiological stress due to injury or infection.

The earliest SII measurement is presumably related to pre-existing chronic inflammatory states, while readings over the subsequent 24-72 hours reflect the immune and vascular systems' ability to adaptively respond to acute injury [12]. Therefore, as can be seen in Figure 1, the temporal trajectory of SII indices, rather than a single or even two measurements, is more informative of the ongoing state of the adaptive immune response following acute injury, whether it is HIBI, as seen here or stroke [16-18], or COVID-19-related brain involvement [12,13,18].

Sequential comparisons of SII measurements also hold the potential to identify novel therapeutic options, such as specific immunomodulatory treatments, especially in challenging areas such as chronic sequelae among survivors of traumatic brain injury and post-stroke patients with various post-stroke neurological syndromes (PSNS) such as depression, apathy, fatigue, and cognitive impairment.

In the current case, a rapid increase in SII titers during the first 24-48 hours, with a failure to return to normal levels (below the threshold of 300, as proposed by Hu et al. [4]), was associated with a poor prognosis, ultimately leading to death. Further research is needed to establish expected SII levels across different age groups to aid ICU clinical decision-making.

## Conclusions

In conclusion, simple, universally available peripheral immune-inflammation biomarkers, such as the serial systemic immune-inflammation indices (SSIIi), have the potential to objectively facilitate clinical diagnosis, monitor disease progression, and predict recovery likelihood from complications, such as those seen in HIBI. While this single case study cannot confirm the definitive value of serial SII measurements, the widespread availability and simplicity of blood count-derived SII warrant further investigation. It is important to recognize the shared pathobiology of neuroinflammation in HIBI, stroke, COVID-19, and related brain involvement. We believe that the exploration of SSIIi in these major public health issues is both necessary and urgent.
